# A perioperative management elective as a transition-to-practice intervention: effects on preparedness, professional identity, and career decision-making

**DOI:** 10.1186/s12909-026-10059-3

**Published:** 2026-08-04

**Authors:** R Seemann, T Gehlen, MQL Bui, JW Kim, RD Hein, U Stöckle, AM Mielke

**Affiliations:** 1 Centrum für Muskuloskeletale Chirurgie, Charité - Universitätsmedizin Berlin, Corporate Member of Freie Universität Berlin, Humboldt-Universität zu Berlin, and Berlin Institute of Health, Charitéplatz 1, Berlin, 10117 Germany; 2 Klinik für Radiologie, Charité - Universitätsmedizin Berlin, Corporate Member of Freie Universität Berlin, Humboldt-Universität zu Berlin, and Berlin Institute of Health, Berlin, Germany; 3 Klinik für Anästhesiologie und Intensivmedizin, Charité - Universitätsmedizin Berlin, Corporate Member of Freie Universität Berlin, Humboldt-Universität zu Berlin, and Berlin Institute of Health, Berlin, Germany

**Keywords:** Perioperative care, Medical education, Elective course, Career orientation, Transition to practice, Surgical education

## Abstract

**Background:**

Perioperative management requires a broad range of knowledge and practical skills essential for early clinical practice, yet perioperative care is often taught in fragmented rather than integrated fashion across undergraduate medical curricula. Beyond technical skills, perioperative care involves anticipatory planning, prioritization, escalation decisions and interdisciplinary coordination. Electives may help bridge this gap but are often assessed primarily by satisfaction or specialty recruitment. We evaluated a perioperative management elective with respect to self-perceived preparedness, educational value across baseline career interests, and its potential role in informed career decision-making.

**Methods:**

We conducted a prospective evaluation of a two-week perioperative elective at a German medical school. Students completed pseudonymized questionnaires before and after the course and at residency entry. Outcomes included self-assessed preparedness across six perioperative domains, perceived readiness for practice, educational value, and career intentions (5-point Likert scales). Paired pre-post comparisons were analyzed using Wilcoxon signed-rank tests. Subgroup analyses explored outcomes by baseline interest in orthopedics and trauma surgery (OTS).

**Results:**

Forty-three students participated; 40 provided paired pre-post data and 22 completed follow-up. Self-perceived preparedness improved significantly across all perioperative domains (all *p* < 0.001). Post-course educational value was high (median 5; 97.6% top-box = Likert 4–5). Gains were independent of baseline OTS interest. Although group-level career interest remained stable, significant within-person changes were observed (*p* = 0.002), indicating bidirectional shifts rather than unidirectional recruitment. At residency start, participants reported high perceived readiness (median 5; 95.5% top-box).

**Conclusions:**

A structured perioperative elective improved perceived preparedness and was valued irrespective of baseline specialty interest. Beyond improvements in self-perceived preparedness, it supported professional identity formation and informed career decision-making. Perioperative teaching may therefore function as a transition-to-practice intervention rather than a specialty recruitment tool, with relevance across specialties.

**Supplementary Information:**

The online version contains supplementary material available at 10.1186/s12909-026-10059-3.

## Introduction

Perioperative management represents a core competency for physicians entering clinical practice and encompasses a broad range of cognitive and procedural responsibilities across the continuum of surgical care [1–3]. These include preoperative evaluation and diagnostic assessment, surgical planning, risk stratification, and perioperative medication management, as well as patient communication and informed consent [1, 3–7]. Intraoperatively, perioperative competence involves adherence to principles of asepsis, correct sterile gowning and behavior in the operating room, and an understanding of surgical workflows [1, 3]. Postoperatively, it extends to wound management, monitoring for complications, coordination of follow-up care, and interprofessional communication [1, 3, 8]. Although some elements of perioperative management are traditionally associated with surgical specialties, many of these competencies are inherently multidisciplinary and essential for physicians across disciplines, including internal medicine, anesthesiology, emergency medicine, and primary care, where perioperative assessment, medication reconciliation, postoperative monitoring, and patient counseling are routinely required [1–7, 9]. Despite their relevance, perioperative knowledge and skills are encountered inconsistently during undergraduate medical education, [10–12 ] often depending on clinical rotations, local teaching culture, and opportunity rather than structured, longitudinal curricula.

Elective courses are frequently used to address gaps in undergraduate medical education, particularly in procedural and practice-oriented domains where content may be insufficiently represented or taught in a fragmented manner across disciplines [13]. However, their evaluation often focuses on short-term satisfaction or on their effectiveness in recruiting students into specific specialties. Such a narrow focus risks overlooking broader educational functions of electives, including the development of professional identity, strengthening of self-efficacy for early clinical responsibilities, and support of informed career decision-making. Importantly, exposure that enables students to critically appraise a specialty and make informed decisions for or against it after structured experience may represent an equally valuable educational outcome.

The educational design of the elective emphasized collaborative case-based learning, supervised clinical participation, and guided reflection, approaches consistent with social constructivist learning theory, which views learning as the construction of knowledge through authentic participation and interaction with more experienced clinicians [14]. In addition, Self-Determination Theory proposes that educational environments supporting learners’ needs for autonomy, competence, and relatedness foster autonomous motivation and facilitate the internalization of learning [15, 16]. These educational concepts informed the design of the elective and provide a theoretical framework for interpreting the observed educational outcomes.

The elective “Prepare for Surgery!” was developed to provide structured teaching in perioperative management within orthopedics and trauma surgery (OTS), while explicitly addressing competencies relevant beyond a single specialty. The course was designed for students both with and without pre-existing interest in surgical careers, aiming to strengthen perioperative preparedness and support the transition from undergraduate training to clinical practice. The present study therefore evaluated the elective beyond simple satisfaction metrics by examining (1) changes in self-reported perioperative preparedness in selected domains, (2) perceived educational value across different baseline career interests, and (3) the persistence of perceived preparedness at the start of residency. Given the educational evaluation design, objective clinical competence was not assessed.

## Methods

### Study design

This study was conducted as a prospective, single-center educational evaluation with repeated measures (pre-course, post-course, and follow-up assessments).

### Setting & participants

Participants were eighth-semester medical students enrolled in a two-week perioperative elective course, “Prepare for surgery!”. During the eighth semester, completion of one elective course was mandatory within the undergraduate curriculum, with students selecting their preferred course from several available elective options. All students enrolled in this elective course during the study period were eligible for participation. Inclusion criteria were enrollment in the elective and provision of informed consent for study participation. There were no additional exclusion criteria. Participation in the study was voluntary and had no influence on students’ grades, course evaluation, or successful completion of the elective.

### Course structure and educational content

The educational goal of the elective was to improve students’ preparedness for perioperative patient management by integrating the knowledge and practical skills required throughout the perioperative pathway. The elective was designed around predefined learning objectives that included structured preoperative assessment and medication management; conducting informed consent discussions for common orthopedic trauma procedures; understanding basic surgical principles, including fracture management, osteosynthesis, and operating room workflow; applying principles of aseptic practice and appropriate operating room behavior; performing basic postoperative wound management; communicating and collaborating effectively within the perioperative team; and reflecting on the professional role of physicians in perioperative patient care.

The two-week elective was structured to mirror the perioperative patient pathway, progressing from preoperative evaluation and decision-making to intraoperative workflows and postoperative care. Teaching formats were deliberately aligned with the clinical context and delivered by an interdisciplinary faculty comprising orthopedics and trauma surgery, anesthesiology, and radiology, with additional interprofessional input from ward and operating room nursing staff and emergency department clinicians.

Week 1 focused on foundational perioperative competencies and clinical decision-making. Core content included case-based seminars on surgical indications, fracture classification, and treatment planning, complemented by imaging interpretation for perioperative decision-making delivered in collaboration with radiology. Interactive sessions addressed preoperative assessment and perioperative medication management, conducted with anesthesiology, as well as structured teaching on surgical informed consent, including legal and communicative principles. Students practiced informed consent using standardized patient scenarios involving distal radius and distal fibula fractures, together with standardized surgical consent forms, to learn the general structure and principles of surgical informed consent applicable to common orthopedic trauma procedures. Together, these formats emphasized anticipatory planning and multidisciplinary decision-making in the preoperative phase.

Week 2 emphasized application, intraoperative workflows, and postoperative care. Students participated in operating room observerships and case-based discussions to contextualize perioperative decision-making within real clinical workflows. Practical training comprised hands-on workshops on surgical instruments and techniques using saw bone models, including standard fixation and stabilization techniques used in OTS. Operating room preparation was addressed through supervised training in surgical hand disinfection, sterile gowning, draping, and patient positioning, as well as appropriate operating room behavior. In addition, students took part in emergency department visitations, during which the workflow surrounding admitted OTS patients was explained and discussed. Postoperative management was addressed through structured instruction in wound management, dressing techniques, and typical postoperative physiotherapeutic care pathways. The elective concluded with an open question-and-answer session involving both junior and senior residents, providing insights into clinical responsibilities, training structures, and everyday professional challenges.

The elective comprised 50 teaching units (45 min each) delivered over two consecutive weeks and enrolled a maximum of 15 students per course. During the study period, the course was offered in three iterations. Foundational seminars and skills laboratory sessions were conducted with the full student cohort, whereas bedside teaching was delivered in groups of six or three students to facilitate active participation. Seminars were led by one faculty member, and bedside teaching was supervised by one faculty member per group. Simulated informed consent sessions and skills laboratory training were facilitated by two faculty members to enable structured observation and feedback. Teaching activities and formative faculty feedback addressed all predefined learning objectives, whereas the questionnaires focused on selected objectives considered suitable for standardized self-assessment. Student learning was assessed throughout the course through supervised observation and formative feedback during practical exercises and simulated patient encounters; no summative examination was conducted. Across both weeks, practice exposure was prioritized, and content sequencing was flexibly adapted to clinical availability while maintaining alignment with the perioperative continuum.

### Data collection instruments

Participants completed standardized questionnaires at three time points:

1. Pre-course (immediately before the elective): demographic information (e.g., age, sex), prior exposure to perioperative teaching and operating room experience, baseline self-assessment of perioperative preparedness, and baseline career intentions.
2. Post-course (immediately after the elective): reassessment of perioperative preparedness, course satisfaction, perceived knowledge gain, perceived educational value, changes in interest in OTS, and updated career intentions.
3. Follow-up (at the beginning of residency): perceived preparedness for perioperative tasks, overall readiness for clinical practice, and retrospective evaluation of the elective’s educational value.

Responses were recorded on 5-point Likert scales, with higher scores indicating greater agreement or perceived preparedness (1 = strongly disagree, 5 = strongly agree). Questionnaires were linked longitudinally using a participant-generated pseudonymized code. The questionnaires, provided in full in the Supplementary Materials (Questionnaires 1–3), were developed specifically for this educational evaluation and aligned with the predefined learning objectives of the elective. They were administered in German, the language of instruction; the English versions provided in the Supplementary Materials are translations for the benefit of an international readership. Prior to implementation, the questionnaires were piloted with five final-year medical students to assess clarity, comprehensibility, and feasibility of completion, resulting in minor wording adjustments. Formal validation and reliability analyses were not performed.

### Outcomes

Primary outcomes were changes in self-assessed perioperative preparedness across six domains:

1. preoperative patient preparation, including medical history taking and basic risk assessment;
2. preoperative medication management, including identification of medications requiring perioperative adjustment;
3. informed consent for standard surgical procedures;
4. surgical hand disinfection according to aseptic standards;
5. sterile gowning and appropriate behavior in the operating room; and
6. basic postoperative wound care, including simple wound management and dressing changes. Secondary outcomes included post-course satisfaction, perceived educational value, perceived preparedness for perioperative management, and changes in career intentions. Follow-up outcomes focused on perceived readiness at the start of residency.

### Statistical analysis

Descriptive statistics are reported as mean ± standard deviation (SD) for normally distributed continuous variables and as counts (%) for categorical variables. Likert-scale outcomes are presented as medians with interquartile ranges (IQR). Paired pre-post comparisons were assessed using Wilcoxon signed-rank tests. Between-group differences by baseline interest in OTS (Likert ≥ 4 vs. < 4) were explored by comparing pre-post change scores using Wilcoxon rank-sum tests. P-values were adjusted for multiple comparisons using the Benjamini-Hochberg false discovery rate (FDR), where applicable. Analyses were performed using R (Version 2024.04.2 + 764, Posit Software, Boston, MA, USA).

## Results

### Participant characteristics

A total of 43 medical students enrolled in the elective “Prepare for Surgery!” and consented to participate in the study. The mean age was 25.9 ± 5.2 years, and 58.5% of participants were female. Most students had prior exposure to the operating room (87.8%), and 65.0% had completed a prior clerkship or internship in a surgical discipline. Baseline interest in OTS was moderate to high (median 4, IQR 1). Baseline characteristics did not differ significantly by sex and are summarized in Table 1.

**Table 1:** Baseline student characteristics

Variable	All (*n* = 43)	Female (*n* = 24)	Male (*n* = 17)	*p*-value
Age, years	25.9 ± 4.2	26.4 ± 6.0	24.5 ± 2.9	0.658
Prior operating room exposure (Yes)	36 (87.8%)	21 (91.3%)	14 (82.4%)	0.634
Prior sterile operating room participation (Yes)	33 (80.5%)	19 (82.6%)	13 (76.5%)	0.702
Prior clerkship or internship in a surgical discipline (Yes)	26 (65.0%)	13 (56.5%)	12 (75.0%)	0.317
Baseline interest in OTS	4.0 (3.0, 5.0)	4.0 (3.0, 5.0)	5.0 (4.0, 5.0)	0.172

Continuous variables are presented as mean ± SD or median (IQR). Categorical variables are presented as n (%). Between-group comparisons by sex were conducted using Wilcoxon rank-sum tests for continuous variables and chi-square or Fisher’s exact tests for categorical variables.

### Pre-post changes in perioperative preparedness

Paired pre-post questionnaire data were available for 40 participants. Significant improvements were observed across all assessed perioperative domains (Table 2).

**Table 2:** Pre-post changes in perioperative preparedness, educational value, and career intentions

	Pre, median (IQR)	Post, median (IQR)	Median change	*p* value	adjusted *p*
Preoperative patient preparation	3 (1.75)	4 (1)	1	< 0.001	< 0.001
Preoperative medication management	2 (2)	4 (1)	2	< 0.001	< 0.001
Informed consent	2 (1.25)	4 (2)	1	< 0.001	< 0.001
Surgical hand disinfection	4 (1)	5 (0)	1	< 0.001	< 0.001
Sterile gowning & OR behavior	4 (2)	5 (0.25)	1	< 0.001	< 0.001
Basic wound care	3 (1)	4 (1)	1	< 0.001	< 0.001

Values are presented as median (IQR). Paired pre-post comparisons were performed using Wilcoxon signed-rank tests. Effect sizes are reported as r. P-values were adjusted for multiple comparisons using the Benjamini-Hochberg false discovery rate. Likert-scale items were rated on a 5-point scale (1 = strongly disagree / not prepared, 5 = strongly agree / very prepared)

Median perceived preparedness for preoperative patient preparation increased from 3 (IQR 1.75) before the course to 4 (IQR 1) after the course (median change + 1, *p* < 0.001). Preparedness for perioperative medication management improved from a median of 2 (IQR 2) to 4 (IQR 1) (median change + 2, *p* < 0.001). Similarly, confidence in conducting informed consent discussions for standard surgical procedures increased from 2 (IQR 1.25) to 4 (IQR 2) (median change + 1, *p* < 0.001). Procedural domains also demonstrated significant gains. Preparedness for surgical hand disinfection increased from a median of 4 (IQR 1) to 5 (IQR 0) (*p* < 0.001), while preparedness for sterile gowning and appropriate operating room behavior improved from 4 (IQR 2) to 5 (IQR 0.25) (*p* < 0.001). Preparedness for basic wound care increased from 3 (IQR 1) to 4 (IQR 1) (*p* < 0.001). All improvements remained statistically significant after adjustment for multiple testing.

Exploratory subgroup analyses revealed no statistically significant differences in pre-post change scores between female and male students across perioperative domains (all adjusted *p* > 0.05).

### Post-course evaluation and perceived educational value

**Fig. 1:**
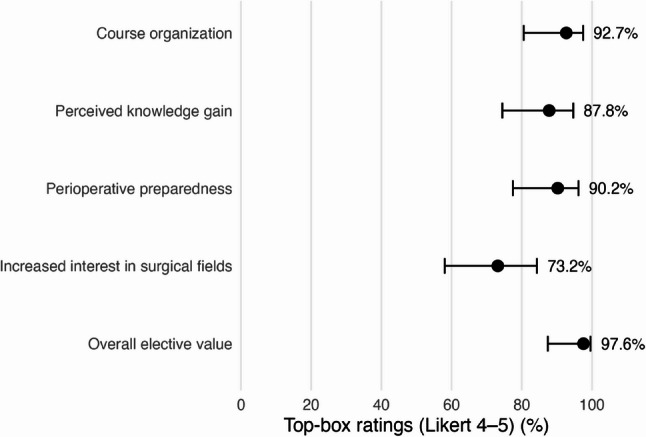
Top-box ratings for post-course evaluation domains Proportion of participants providing top-box ratings (Likert scale 4 - 5) for post-course evaluation domains, displayed with 95% Wilson confidence intervals. Percentages and sample sizes are shown. Likert scale ranged from 1 = strongly disagree to 5 = strongly agree

Post-course evaluations were completed by 40 participants. Overall satisfaction with course organization was high, with a median rating of 5 (IQR 1); 92.7% of students rated this outcome in the top-box range (Likert 4–5) (Fig. 1). Perceived knowledge gain was rated with a median of 4 (IQR 1), with 87.8% of participants reporting top-box agreement. Perceived preparedness for perioperative management was rated with a median of 5 (IQR 1).

Students also reported a positive effect of the elective on their interest in surgical fields, with a median rating of 4 (IQR 2); 73.2% of participants indicated a top-box increase in interest. The perceived educational value of the elective was rated particularly high, reaching a median of 5 (IQR 0), with 97.6% of students assigning top-box ratings.

**Fig. 2:**
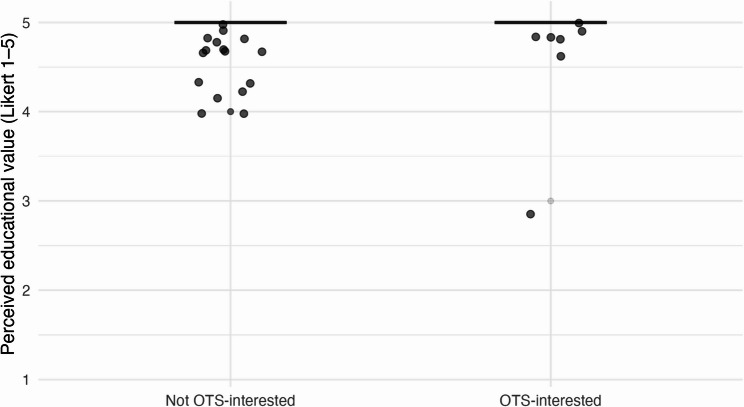
Perceived educational value of the elective by baseline interest in orthopedics and trauma surgery Box plots show post-course ratings of the perceived educational value of the elective on a 5 - point Likert scale (1 = strongly disagree, 5 = strongly agree), stratified by baseline interest in orthopedics and trauma surgery (OTS). Baseline OTS interest was defined as Likert scores of 4 - 5, while non-OTS interest was defined as Likert scores of 1 - 3. Boxes represent the median and interquartile range, whiskers indicate 1.5x the interquartile range, and individual points represent single participants

Perceived educational value remained high irrespective of baseline interest in OTS, with students without baseline OTS interest reporting post-course value ratings comparable to those of OTS-interested students (Fig. 2).

### Career intentions and orientation

**Fig. 3:**
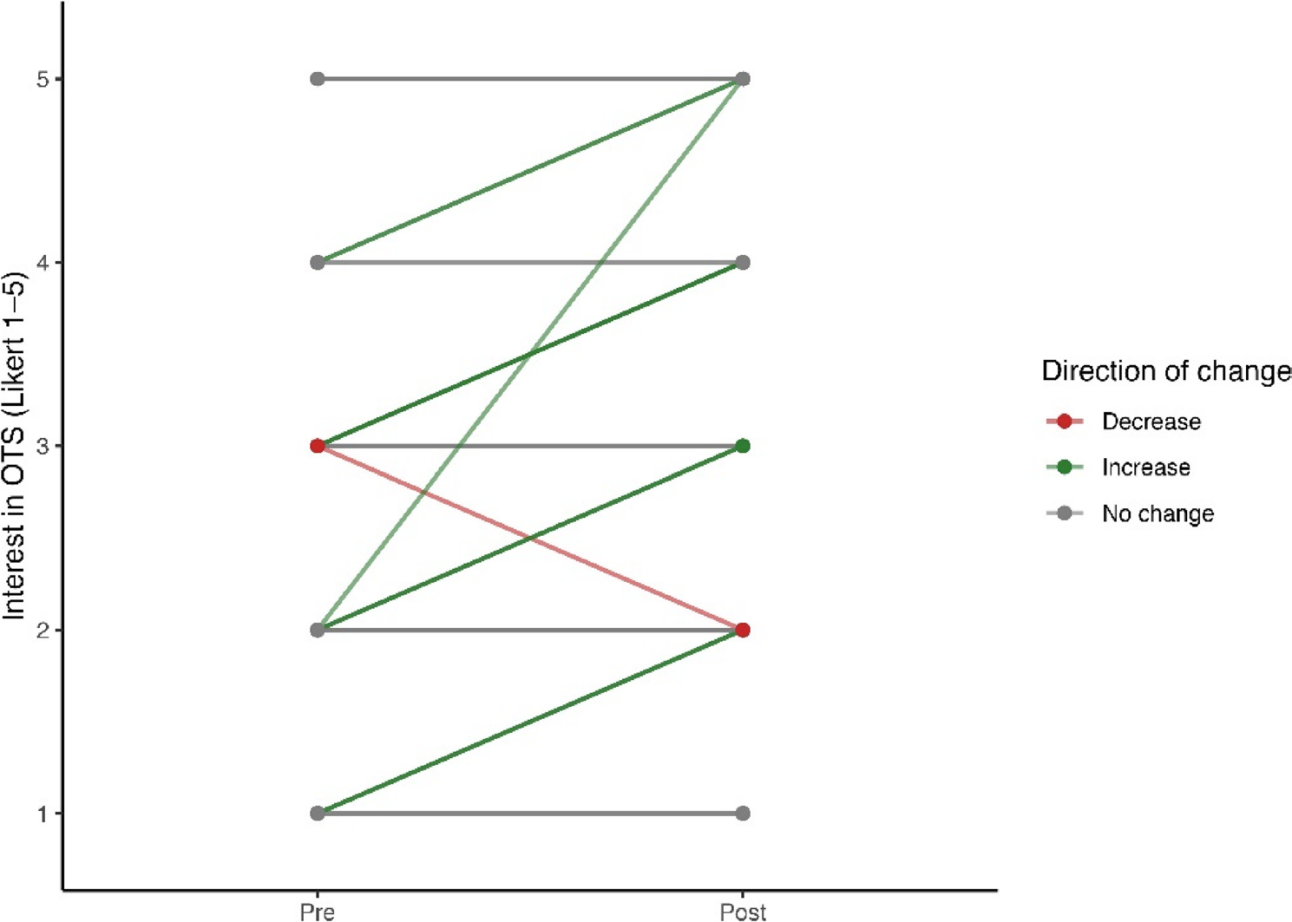
Individual pre-post changes in interest in orthopedics and trauma surgery Individual pre-post trajectories of OTS interest (*n* = 40). Despite an unchanged group median, within-person changes were significant (Wilcoxon signed-rank test, *p* = 0.002), demonstrating heterogeneous bidirectional shifts

Paired pre-post data on career intentions were available for 40 participants. Median interest in pursuing a residency in OTS remained 3 (IQR 2) both before and after the elective. Although median interest in OTS remained unchanged at the group level, paired analysis revealed significant within-person changes in interest scores (*p* = 0.002). Visualization of individual trajectories demonstrating bidirectional within-person changes that were not apparent in the group median is shown in Fig. 3, highlighting the heterogeneity of career orientation shifts. In contrast, no statistically significant pre-post difference was observed for interest in pursuing a residency in another surgical field (median 3 [IQR 2] both pre- and post-course; *p* = 0.697). Median interest in pursuing a residency in a non-surgical field also remained unchanged following the elective (median 2 [IQR 2.75] both before and after the course; *p* = 0.824).

Transition analysis demonstrated that all students who were OTS-oriented at baseline (*n* = 13; Likert ≥ 4) remained OTS-oriented after completing the elective (Supplementary Fig. 1). Among students without baseline OTS orientation (*n* = 27), five students (18.5%) shifted toward OTS orientation following the course. However, McNemar’s test did not demonstrate a statistically significant net directional change (*p* = 0.074).

Of these transitioning students, 4 (80.0%) had reported a baseline interest in another surgical specialty, while one student (20.0%) had reported a baseline preference for a non-surgical field. Free-text responses among transitioning students indicated baseline interests in pediatric surgery, neurosurgery, and plastic surgery (each *n* = 1); one student did not specify the surgical discipline. One transitioning student reported anesthesiology, which was classified as the non-surgical specialty. In contrast, among students who did not transition toward OTS, baseline interests were evenly distributed between other surgical fields (54.5%) and non-surgical fields (45.5%). Exploratory analyses did not identify a statistically significant association between baseline interest in another surgical field and transition toward OTS (Fisher’s exact test, *p* = 0.342), nor between baseline non-surgical orientation and transition (*p* = 0.342).

Additional exploratory analyses showed that neither lower baseline perioperative preparedness (*p* = 0.889) nor greater pre-post learning gains across perioperative domains (*p* = 0.808) were significantly associated with transition toward OST orientation.

### Follow-up at residency start

Follow-up data at the beginning of residency were available for 22 participants. Because follow-up was scheduled for the expected beginning of residency, not all participants had reached this time point when the analyses were performed. Reasons for non-response among those contacted were not assessed. Perceived readiness for residency was high, with a median rating of 5 (IQR 0), and 95.5% of respondents reported top-box ratings. Among these participants, 13 had reported high post-course interest in OTS (Likert 4–5). Of these, 11 (84.6%) subsequently entered OTS residency, while one (7.7%) entered another surgical specialty (neurosurgery) and one (7.7%) entered a non-surgical specialty (anesthesiology), corresponding to 92.3% entering a surgical specialty overall.

## Discussion

This study evaluated a structured perioperative management elective for undergraduate medical students with the explicit aim of moving beyond satisfaction-based course evaluation toward understanding its educational relevance for clinical preparedness, professional identity formation, and informed career decision-making. Consistent improvements in perceived preparedness across perioperative domains suggest that structured teaching can bridge the gap between theoretical knowledge and the practical responsibility encountered during the transition into residency.

Early postgraduate surgical training requires junior physicians to assume increasing autonomy in perioperative care, going beyond technical execution to include cognitive and leadership competencies. Contemporary models of perioperative care emphasize proactive, physician-led oversight of the perioperative trajectory, with responsibilities for anticipatory planning, escalation decisions, prioritization, and coordination across teams [2]. The gains observed in our study align with educational evidence suggesting that confidence and preparedness developed through structured training may facilitate the transition to early clinical practice, whereas objective competence has been associated with safe early autonomy and reduced supervision needs [17, 18].

Surgery represents a common healthcare event across the lifespan: approximately one in nine individuals in the United States undergoes at least one surgical procedure annually, with rates approaching one in five among adults aged 65 years and older [19]. Globally, over 300 million surgical procedures are performed each year [20]. As a result, perioperative considerations affect a substantial share of inpatient care, across both surgical and non-surgical services. Physicians across multiple specialties are therefore routinely involved in perioperative assessment, coordination, and follow-up, supporting perioperative management as core educational content rather than optional specialty exposure.

Perioperative teaching appears to contribute to professional identity formation, a central developmental process in undergraduate medical education. Through authentic participation and reflection, learners internalize professional role expectations [21, 22]. In our study, this was reflected in significant within-person changes in career interest despite an unchanged group median (Fig. 3), highlighting the heterogeneity of individual decision-making trajectories. The bidirectional nature of these shifts suggests that the elective did not function as a simple recruitment mechanism but rather as a reflective space in which students reassessed alignment with surgical and non-surgical pathways. At this advanced stage of training (8th semester of a 12-semester medical curriculum), when students must increasingly orient toward residency applications during the subsequent practical year (11th -12th semesters), structured exposure may support informed, experience-based career clarification rather than assumption-driven choices.

In a recent large cross-sectional survey of medical students in Germany and Austria, Ketzer et al. 23]. reported that interest in OTS declines over medical school among female students, and identified work environment/culture and practical clinical experiences as key determinants of specialty choice. Perceived barriers included active discouragement, frequently reported to originate from OTS physicians themselves. These data underscore why structured, supportive clinical exposure matters and provide a rationale for focusing not only on knowledge and skill acquisition, but how students are integrated into clinical work.

In line with this, educational research suggests that learning outcomes in clinical electives are driven less by baseline interest than by the perceived educational climate. Factors such as supervision quality and opportunities for meaningful participation have been shown to predict engagement and learning more strongly than prior career intentions [24]. Self-determination theory posits that environments fostering autonomy, competence, and relatedness can convert situational engagement into meaningful learning, even in the absence of strong intrinsic interest [15, 16]. While increasing interest may be a secondary benefit of specialty-specific electives, the primary educational aim of this course was to enhance perioperative preparedness and support informed career decision-making. In this context, learning gains were independent of baseline specialty interest, and transitions toward OTS occurred predominantly among students with prior surgical interest, suggesting informed differentiation within related fields rather than broad recruitment into the specialty.

The follow-up findings at the beginning of residency suggest that participants perceived these effects to be sustained over time. Participants reported sustained preparedness at residency entry, and early specialty choices were largely concordant with post-course intentions. Although follow-up is ongoing, these preliminary data suggest that structured perioperative teaching may support experience-based and internally grounded career decisions, rather than transient interest shifts driven by short-term exposure [25].

Taken together, these findings highlight the potential value of structured perioperative education within undergraduate medical curricula. Consideration should be given to more coherent integration of perioperative management across existing curricula, rather than addressing its components only through fragmented teaching across disciplines. Given the interdisciplinary nature of perioperative care, such integration would require collaboration among multiple specialties and may enhance individual preparedness while promoting interprofessional collaboration and patient safety, competencies central to contemporary hospital practice [26].

### Limitations

This study has several limitations. Outcomes were primarily based on self-reported measures and may not directly reflect objective clinical competence or performance. The elective was evaluated without a parallel control group. Participation in the study was voluntary, and enrollment in this elective was based on students’ selection of one of several mandatory elective options within the curriculum. Consequently, participants may have differed from the general student population in their baseline interest in perioperative care or surgery, introducing selection bias and limiting the generalizability of the findings. Participants were eighth-semester students within a model curriculum that provides substantial early clinical exposure. Although this timing allowed evaluation at a meaningful stage prior to residency entry, career decisions may continue to evolve during subsequent clinical semesters until graduation (12th semester).

Although the questionnaires were piloted for clarity, some items relied on educational context established during the course or used context-dependent terminology, which may have allowed variation in interpretation when considered in isolation. Future studies should further refine questionnaire wording and undertake formal validation.

Lastly, follow-up data at residency start were available for a limited proportion of participants and should therefore be interpreted as exploratory. While these data provide preliminary insight into longer-term perceptions of preparedness, the durability of effects beyond the early postgraduate phase remains to be established.

## Conclusion

This study highlights perioperative management teaching as an educational domain that extends beyond procedural skill acquisition. Structured exposure to perioperative workflows, decision-making, and interprofessional coordination appears to support early clinical preparedness, professional identity formation, and informed career decision-making.

Rather than functioning primarily as a recruitment mechanism, the elective facilitated clarification of career alignment, including informed decisions both for and against surgical pathways. These findings support the integration of perioperative management as a longitudinal, interdisciplinary component of undergraduate medical curricula, with relevance for the transition to clinical practice across specialties.

## Supplementary Information

Supplementary Material 1.

## Data Availability

The datasets used and analyzed during the current study are available from the corresponding author on reasonable request.
