# Supplementary material for: A perioperative management elective as a transition-to-practice intervention: effects on preparedness, professional identity, and career decision-making
Source: BMC Med Educ. 2026 Aug 4;26:1244. doi: 10.1186/s12909-026-10059-3 (PMC13435946; doi:10.1186/s12909-026-10059-3)
Supplement: Supplementary file 1 [file 12909_2026_10059_MOESM1_ESM.docx]

### **Supplementary Materials**

### **Questionnaire 1. Pre-Intervention Questionnaire**

*Please generate a personal code word using the* ***first 2 letters of your mother's first name****, the* ***first 2 letters of your father's first name****, and the* ***last 2 letters of your place of birth****.*

Code word: ____________________________

**Training Status** Age: ______ Gender: m / f / d

**1.** During my medical studies, I attended course(s) on the topic of "perioperative management."

☐ Strongly disagree (1) ☐ Disagree (2) ☐ Neither agree nor disagree (3) ☐ Agree (4) ☐ Strongly agree (5)

**2.** If yes, in what format? ☐ Lecture ☐ Seminar ☐ Clinical skills training ☐ Other: _______

**3.** Prior to starting this elective, I had already completed a clinical placement/internship in trauma surgery or orthopedics. ☐ Yes    ☐ No

**4.** Prior to this elective, I had already attended/observed a surgical procedure. ☐ Yes    ☐ No

**4a.** I was able to perform a sterile surgical scrub and stand sterile at the operating table.    ☐ Yes    ☐ No

**5.** If yes, in what context? ☐ Nursing internship ☐ Clinical placement, semester: _______

☐ Employment as: _______ ☐ Other: _______

**Subjective Prior Knowledge**

**6.** I know what basic preoperative patient preparation involves.

☐ Strongly disagree (1) ☐ Disagree (2) ☐ Neither agree nor disagree (3) ☐ Agree (4) ☐ Strongly agree (5)

**6a.** I feel prepared to review a patient's preoperative medication and have a basic understanding of which drug classes need to be paused or adjusted and within what timeframes.

☐ Strongly disagree (1) ☐ Disagree (2) ☐ Neither agree nor disagree (3) ☐ Agree (4) ☐ Strongly agree (5)

**6b.** I feel prepared overall to conduct a surgical informed consent discussion for a standard procedure under supervision.

☐ Strongly disagree (1) ☐ Disagree (2) ☐ Neither agree nor disagree (3) ☐ Agree (4) ☐ Strongly agree (5)

**7.** I feel prepared to independently perform a surgical hand disinfection.

☐ Strongly disagree (1) ☐ Disagree (2) ☐ Neither agree nor disagree (3) ☐ Agree (4) ☐ Strongly agree (5)

**8.** I feel prepared overall to dress in sterile attire in the operating room and know which sterile working rules must be followed.

☐ Strongly disagree (1) ☐ Disagree (2) ☐ Neither agree nor disagree (3) ☐ Agree (4) ☐ Strongly agree (5)

**Expectations for the Elective / Future Career Plans**

**9.** Prior to starting this elective, I was already interested in trauma surgery / orthopedics.

☐ Strongly disagree (1) ☐ Disagree (2) ☐ Neither agree nor disagree (3) ☐ Agree (4) ☐ Strongly agree (5)

**10.** In my view, this elective will be a valuable addition to my current and future medical training.

☐ Strongly disagree (1) ☐ Disagree (2) ☐ Neither agree nor disagree (3) ☐ Agree (4) ☐ Strongly agree (5)

**11.** It is likely that after receiving my medical license I will pursue specialty training in trauma surgery / orthopedics.

☐ Strongly disagree (1) ☐ Disagree (2) ☐ Neither agree nor disagree (3) ☐ Agree (4) ☐ Strongly agree (5)

**12.** It is likely that after receiving my medical license I will pursue specialty training in another surgical field.

☐ Strongly disagree (1) ☐ Disagree (2) ☐ Neither agree nor disagree (3) ☐ Agree (4) ☐ Strongly agree (5)

**12a.** Which one? _______

**13.** It is likely that after receiving my medical license I will pursue specialty training in a non-surgical field.

☐ Strongly disagree (1) ☐ Disagree (2) ☐ Neither agree nor disagree (3) ☐ Agree (4) ☐ Strongly agree (5)

**13a.** Which one? _______

**14.** The following knowledge and skills I would like to learn/deepen through this elective:

### **Questionnaire 2. Post-Intervention Questionnaire**

*Please enter the code word you previously generated using the* ***first 2 letters of your mother's first name****, the* ***first 2 letters of your father's first name****, and the* ***last 2 letters of your place of birth****.*

Code word: ____________________________

**Training Status** Age: ______ Gender: m / f / d

**Subjective Knowledge Gain / Attitudes**

**1.** I know what basic preoperative patient preparation involves.

☐ Strongly disagree (1) ☐ Disagree (2) ☐ Neither agree nor disagree (3) ☐ Agree (4) ☐ Strongly agree (5)

**1a.** I feel prepared to review a patient's preoperative medication and have a basic understanding of which drug classes need to be paused or adjusted and within what timeframes.

☐ Strongly disagree (1) ☐ Disagree (2) ☐ Neither agree nor disagree (3) ☐ Agree (4) ☐ Strongly agree (5)

**1b.** I feel prepared overall to conduct a surgical informed consent discussion for a standard procedure under supervision.

☐ Strongly disagree (1) ☐ Disagree (2) ☐ Neither agree nor disagree (3) ☐ Agree (4) ☐ Strongly agree (5)

**2.** I feel prepared to independently perform a surgical hand disinfection.

☐ Strongly disagree (1) ☐ Disagree (2) ☐ Neither agree nor disagree (3) ☐ Agree (4) ☐ Strongly agree (5)

**3.** I feel prepared overall to dress in sterile attire in the operating room and know which sterile working rules must be followed.

☐ Strongly disagree (1) ☐ Disagree (2) ☐ Neither agree nor disagree (3) ☐ Agree (4) ☐ Strongly agree (5)

**Elective Evaluation / Future Career Plans**

**4.** Overall, I am satisfied with the delivery of the elective "Prepare for Surgery!".

☐ Strongly disagree (1) ☐ Disagree (2) ☐ Neither agree nor disagree (3) ☐ Agree (4) ☐ Strongly agree (5)

**5.** Overall, I am satisfied with my individual knowledge gain.

☐ Strongly disagree (1) ☐ Disagree (2) ☐ Neither agree nor disagree (3) ☐ Agree (4) ☐ Strongly agree (5)

**6.** After this elective, I feel better prepared overall for perioperative management.

☐ Strongly disagree (1) ☐ Disagree (2) ☐ Neither agree nor disagree (3) ☐ Agree (4) ☐ Strongly agree (5)

**7.** My interest in trauma surgery / orthopedics has increased through this elective.

☐ Strongly disagree (1) ☐ Disagree (2) ☐ Neither agree nor disagree (3) ☐ Agree (4) ☐ Strongly agree (5)

**8.** In my view, this elective is a valuable addition to my current and future medical training.

☐ Strongly disagree (1) ☐ Disagree (2) ☐ Neither agree nor disagree (3) ☐ Agree (4) ☐ Strongly agree (5)

**9.** It is likely that after receiving my medical license I will pursue specialty training in trauma surgery / orthopedics.

☐ Strongly disagree (1) ☐ Disagree (2) ☐ Neither agree nor disagree (3) ☐ Agree (4) ☐ Strongly agree (5)

**10.** It is likely that after receiving my medical license I will pursue specialty training in another surgical field.

☐ Strongly disagree (1) ☐ Disagree (2) ☐ Neither agree nor disagree (3) ☐ Agree (4) ☐ Strongly agree (5)

**10a.** Which one? _______

**11.** It is likely that after receiving my medical license I will pursue specialty training in a non-surgical field.

☐ Strongly disagree (1) ☐ Disagree (2) ☐ Neither agree nor disagree (3) ☐ Agree (4) ☐ Strongly agree (5)

**11a.** Which one? _______

**12.** What I particularly enjoyed:

**13.** My wishes, suggestions, or recommendations for improvement:

**Supplementary figure 1.** Transition of career orientation toward orthopedics and trauma surgery

*Transition matrix illustrating changes in career orientation toward OTS before and after completion of the elective. Career orientation was dichotomized based on Likert-scale ratings (Likert ≥ 4 indicating OTS orientation). Values represent counts and row percentages (n=40).*
